# Investigating the Interplay of Toxic Metals and Essential Elements in Liver Disease

**DOI:** 10.3390/ijerph21060762

**Published:** 2024-06-12

**Authors:** Aderonke Gbemi Adetunji, Emmanuel Obeng-Gyasi

**Affiliations:** 1 Department of Built Environment, North Carolina A&T State University, Greensboro, NC 27411, USA; 2 Environmental Health and Disease Laboratory, North Carolina A&T State University, Greensboro, NC 27411, USA

**Keywords:** toxic metals, essential elements, liver dysfunction, NAFLD, lead exposure, manganese, selenium, multivariable regression, Bayesian Kernel Machine Regression, environmental health

## Abstract

Liver diseases, including non-alcoholic fatty liver disease (NAFLD), are a growing global health issue. Environmental exposure to toxic metals can harm the liver, increasing the risk of NAFLD. Essential elements are vital for liver health, but imbalances or deficiencies can contribute to the development of NAFLD. Therefore, understanding the interplay between toxic metals and essential elements in liver disease is important. This study aims to assess the individual and combined effects of toxic metals (lead(Pb), cadmium (Cd), mercury (Hg)), and essential elements (manganese and selenium) on the risk of liver disease. Methods: We assessed the individual and combined effects of Pb, Cd, Hg, manganese (Mn), and selenium (Se) on liver disease risk using data from the National Health and Nutrition Examination Survey between 2017 and 2018. We performed descriptive statistics and linear regression analysis and then utilized Bayesian Kernel Machine Regression (BKMR) techniques such as univariate, bivariate, and overall effect analysis. BKMR enabled the assessment of non-linear exposure–response functions and interactions between metals and essential elements. Posterior Inclusion Probabilities (PIPs) were calculated to determine the importance of each metal and essential element in contributing to liver disease. Regarding our study results, the regression analysis of liver injury biomarkers ALT, AST, ALP, GGT, total bilirubin, and the FLI—an indicator of NAFLD—with toxic metals and essential elements, adjusting for covariates such as age, sex, BMI, alcohol consumption, ethnicity, income, and smoking status, demonstrated the differential effects of these contaminants on the markers of interest. Our BKMR analysis provided further insights. For instance, the PIP results underscored Pb’s consistent importance in contributing to liver disease (PIP = 1.000), followed by Hg (PIP = 0.9512), Cd (PIP = 0.5796), Se (PIP = 0.5572), and Mn (PIP = 0.4248). Our univariate analysis showed a positive trend with Pb, while other exposures were relatively flat. Our analysis of the single-variable effects of toxic metals and essential elements on NAFLD also revealed that Pb significantly affected the risk of NAFLD. Our bivariate analysis found a positive (toxic) trend when Pb was combined with other metals and essential elements. For the overall exposure effect of exposure to all the contaminants together, the estimated risk of NAFLD showed a steady increase from the 60th to the 75th percentile. In conclusion, our study indicates that Pb exposure, when combined with other toxic metals and essential elements, plays a significant role in bringing about adverse liver disease outcomes.

## 1. Introduction

Liver diseases are pathological states that impair the hepatic system’s structural integrity and physiological functionality [1]. The types of liver diseases include viral hepatitis, non-alcoholic fatty liver disease (NAFLD), alcoholic liver disease, cirrhosis, hepatocellular carcinoma (HCC), cholestatic liver diseases, autoimmune liver diseases, and metabolic liver diseases [1]. However, the most prevalent liver diseases include viral hepatitis (hepatitis B and C), alcoholic liver disease, and NAFLD [1].

Liver diseases represent a significant global public health challenge, impacting millions of individuals across various populations worldwide [2]. They also place a significant healthcare and economic burden on societies due to the expensive cost of diagnosis, treatment, and liver transplantation. Globally, liver diseases account for about 2 million deaths yearly. According to the World Health Organization (WHO) 2024 Global Hepatitis Report, the mortality attributed to viral hepatitis is on the rise. It ranks as the second most significant infectious cause of death worldwide, resulting in 1.3 million deaths annually [2]. A recent meta-analysis reports a global prevalence of NAFLD at 25.2%, with the highest rates observed in the Middle East and South America and the lowest in Africa [3]. In the United States, NAFLD impacts approximately 80–100 million individuals, making it the leading cause of chronic liver disease [4].

Liver diseases are induced by a multitude of risk factors, encompassing age, alcohol consumption, tobacco use, environmental exposures, dietary habits, genetic predispositions, specific infectious agents, and underlying chronic health conditions. Chronic and excessive alcohol intake notably contributes to the pathogenesis of various hepatic disorders, primarily through the metabolic processes associated with its breakdown [1,5]. Exposure to environmental pollutants, including persistent organic pollutants (POPs) and heavy metals, has been associated with a higher likelihood of developing NAFLD [6]. These pollutants have the potential to disturb regular metabolic functions and elicit oxidative stress and inflammation in the liver [6]. Consumption of diets high in saturates and trans-fat, added sugar, and refined carbohydrates has been linked to the constant increased risk of NAFLD [7]. Consumption of these deleterious dietary components can result in weight gain, obesity, and insulin resistance through increased lipogenesis, impaired fatty acid oxidation, and the promotion of oxidative stress and inflammation in the liver [7,8]. The development of obesity also contributes to NAFLD through insulin resistance, increased oxidative stress and inflammation and altered gut microbiome [8].

The liver is a critical organ involved in the metabolism and homeostasis of essential elements necessary for proper functioning [9]. The liver, for example, regulates zinc homeostasis in the body. Zinc is essential for the function of numerous enzymes, maintaining cell membrane integrity, and regulating gene expression [10,11]. Imbalances and deficiencies in the levels of essential elements can affect the liver health negatively. Careful monitoring and management of these elements are crucial in preventing and treating liver diseases.

Toxic or heavy metals are naturally occurring metallic elements that have been shown to adversely affect environmental and health systems [12]. Examples of such metals include Pb, Hg, arsenic (As), Cd, and chromium. Sources of exposure include industrial pollution, vehicular emissions, occupational activities, agricultural practices, and contamination of food and water sources. These metals can enter the human body through ingestion, inhalation, and dermal absorption. The chronic accumulation of these metals can lead to hepatic toxicity.

The liver is essential for the regulation of essential metal homeostasis and the metabolism and excretion of toxic metals in the body [9]. Essential elements are crucial cofactors for numerous liver enzymes, which are vital in maintaining proper liver function [12]. However, the presence of toxic metals can disrupt this delicate homeostasis by interfering with the absorption, distribution, and utilization of essential elements [13]. The disturbance of this homeostasis can contribute to oxidative stress, inflammation, and the onset of various liver diseases [14,15]. Studies have explored the relationship between toxic metals and liver health. A cross-sectional cohort study by Cave and colleagues aimed to investigate the potential association between environmental pollutants and increased serum alanine aminotransferase (ALT) activity among U.S. adults. The study focused on adult participants without viral hepatitis, hemochromatosis, or alcoholic liver disease, drawn from the National Health and Nutrition Examination Survey (NHANES) for the years 2003–2004. The findings revealed that exposures to polychlorinated biphenyls, Pb, and Hg were linked to unexplained elevations in ALT, serving as a proxy marker for NAFLD [16]. In another study aiming to assess the relationship between creatinine-corrected urinary Cd levels and various liver-related outcomes in the general U.S. population, data from 12,732 adults who participated in the Third National Health and Nutrition Examination Survey conducted during 1988–1994 (NHANES III) were analyzed. They examined the association between individuals in the highest quartile for urinary Cd levels and hepatic necroinflammation, non-alcoholic fatty liver disease (NAFLD), and non-alcoholic steatohepatitis (NASH). The findings revealed that environmental exposure to Cd was linked to hepatic necroinflammation, NAFLD, and NASH among men and hepatic necroinflammation among women. Moreover, individuals in the highest quartile of creatinine-corrected urinary Cd had more than a threefold increased risk of liver disease mortality [17].

Existing research has elucidated the relationship between exposure to toxic metals and the risk of liver diseases. However, the synergistic effects of toxic metals in conjunction with essential elements have not been comprehensively explored. Understanding the complex interactions between essential elements and toxic metals in the liver is crucial for devising new and effective strategies to mitigate liver toxicity and associated diseases. Therefore, this study aims to fill a significant gap in the literature by assessing the combined impacts of toxic metals (Pb, Cd, and Hg) and essential elements (Mn, Se) on the risk of liver disease risk.

Traditional analytical approaches for assessing the environmental impacts of heavy metals on NAFLD often consider each pollutant individually. However, real-world exposure involves mixtures of the pollutants, necessitating advanced statistical methods to evaluate their combined health effects. One such prominent method in environmental health research is BKMR [18]. BKMR is an innovative statistical method that is utilized to address the challenges of multi-pollutant exposure analysis [19]. It enables the assessment of the health effects of pollutant mixtures, taking into account potential interactions and synergistic effects among different pollutants. BKMR is especially beneficial for handling highly correlated exposures and offers insights into both the combined and individual effects of each component in the mixture [19,20]. BKMR’s flexibility in modeling non-linear relationships and varying sensitivities to different exposure levels is essential, given the complexity of biological responses to toxicants [21]. This approach not only evaluates the combined effect of these metals on liver disease but also identifies the specific contribution of each metal, thereby enhancing our understanding of their individual roles in NAFLD.

## 2. Methodologies

### 2.1. Study Design

For this study, we utilized data from the National Health and Nutrition Examination Survey (NHANES) for the years 2017–2018 as the survey contained all the variables needed for our study and was the most recent edition published. NHANES is a cross-sectional, multiphase survey designed to assess the nutritional status and overall health of a nationally representative sample of non-institutionalized individuals in the United States. This dataset represents a sample of non-institutionalized individuals residing in all 50 U.S. states and the District of Columbia. Collected by the U.S. Centers for Disease Control and Prevention (CDC), the data are available in two-year cycles and consist of multi-year, stratified, multi-stage, and clustered samples. Informed consent was obtained from all participants through a physical examination and an interview. Blood was drawn from the participants, and the samples were sent to a laboratory for analysis. The Institutional Review Board approved the survey protocols at the National Center for Health Statistics (NCHS), part of the Centers for Disease Control and Prevention (CDC). In addition, demographic factors were collected. Specifically, variables such as age, sex, and ethnicity were recorded. These variables were gathered through household interviews conducted using a Computer-Assisted Personal Interview (CAPI) system, ensuring accurate and reliable data collection. Respondents provided their demographic information during these interviews, which were administered in multiple languages to accommodate diverse participants.

The serum levels of liver enzymes, including AST, ALT, GGT, ALP, and Total Bilirubin, were quantified using the Roche Cobas 6000 (c501 module) analytical method. These assays were conducted at the University of Minnesota Advanced Research and Diagnostic Laboratory (ARDL) under the auspices of the National Center for Environmental Health (NCEH) of the CDC’s Division of Laboratory Sciences. Detailed specimen collection, storage, and analysis protocols are outlined in the NHANES Laboratory Procedures Manual [22,23,24,25,26].

### 2.2. US-FLI and NAFLD

US-FLI was utilized for NAFLD prediction due to the absence of abdominal ultrasound results in the dataset. It has been validated as a reliable predictor of NAFLD within the United States. US-FLI is computed through a logistic regression model, incorporating BMI, waist circumference, GGT, and triglycerides (TGs), as depicted in the equation below. In our study, the US-FLI was calculated as follows:

FLI = exp(A)/(1 + exp(A)) × 100

where

A = 0.953 × log (TG) + 0.139 × BMI + 0.718 × log (GGT) + 0.053 × waist circumference − 15.745

Additionally, log(.) is the natural logarithm.

### 2.3. Variables and Covariates for Model Adjustment

In our study, the liver injury enzymes and US-FLI served as the outcome variable, while essential elements (Mn and Se) and toxic metals (Pb, Cd, and Hg) acted as predictor variables (exposures). Covariates utilized for model adjustment encompassed age, sex, BMI, smoking status, alcohol consumption, ethnicity, and income. Potential covariates were selected based on prior research investigating the impact of environmental pollutants like metals on liver disease risk.

### 2.4. Statistical Analysis

#### 2.4.1. Descriptive Statistics and Regression Analysis

In our study, we employed descriptive statistics to characterize the critical study variables in the dataset, including sex, age, and ethnicity. Linear regression analysis was subsequently conducted to examine the relationship between the outcome variables, predictive variables, and the covariates in the dataset.

#### 2.4.2. Bayesian Kernel Machine Regression (BKMR)

We employed Bayesian Kernel Machine Regression (BKMR) utilizing the Markov Chain Monte-Carlo (MCMC) sampling approach, as described by Bobb et al. [20]. The analytical process involved 5000 iterations. Our BKMR investigation produced Posterior Inclusion Probabilities (PIPs), which are crucial in determining the influence of metals and essential elements. These PIPs, ranging from 0 to 1, aid in determining the relative importance of each metal and essential element.

To enhance our comprehension of the interaction among these toxic metals, essential elements, and the outcome of interest, we computed high-dimensional exposure–response functions, represented as h(z), at various intervals. This was accomplished while maintaining the other influencing variables constant at their median values. The graphical interpretation function of the BKMR model played a significant role in our analysis. This feature allowed for a comparative examination of the effects of toxic metals and essential elements, both collectively and individually. It compared the outcome observed at certain exposure percentiles to those at median exposure levels. Additionally, it highlighted the unique association between each toxic metal and essential element with NAFLD while accounting for constant median values of other exposures.

In this study, the significance level was set at 0.05. Our study’s analysis was conducted with R (version 4.2.3; R Foundation for Statistical Computing, Vienna, Austria) [27].

## 3. Results

### 3.1. Descriptive Analysis of Sex, Ethnicity, and Age

Table 1a displays that the dataset consists of males with an average age of 37 years and females with an average age of 39 years. As shown in Table 1b, the ethnic composition of the dataset is composed of participants from diverse racial/ethnic groups ranging from Mexican American, other Hispanic, Non-Hispanic White, Non-Hispanic Black, Non-Hispanic Asian, and other Race—including Multi-Racial—with approximately average ages of 29, 34, 42, 36, 38, and 33 years, respectively. These data indicate that non-Hispanic whites have the highest average age, suggesting they represent an older demographic within the dataset. In contrast, Mexican Americans have the lowest average age, indicating a younger demographic. The 95% confidence intervals provide a measure of reliability for these estimates, with some groups showing greater variability (e.g., Other Race—Including Multi-Racial) compared to others.

Indeed, in the context of NHANES, the Linearized Standard(std) Error and “95% Confidence Interval” are crucial statistical measures for accurate data interpretation. The linearized std error measures variability or uncertainty in an estimate adjusted for NHANES’ complex survey design. It indicates the precision of an estimate, where smaller errors mean more precise estimates and larger errors indicate greater variability. The 95% confidence interval is a range that likely contains the true population parameter with 95% certainty. This interval reflects the reliability of an estimate, with narrow intervals suggesting precise estimates and wide intervals indicating more uncertainty. Together, these measures are vital for assessing the precision and reliability of results.

#### Correlation between Variables of Interest

**Figure 1:**
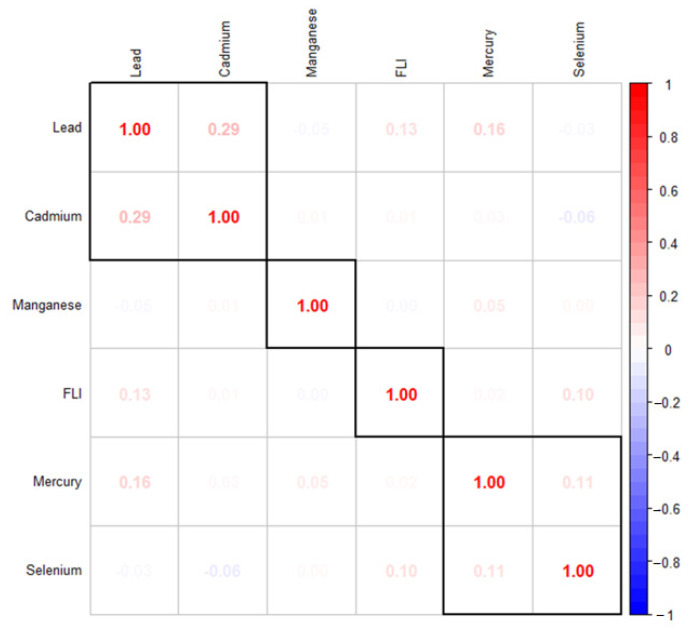
The Spearman correlation analysis among the study’s exposure variables.

Figure 1 explores the correlation between the study variables of interest. It shows that metals, US-FLI, and essential elements show a varying correlation individually among themselves with toxic metals generally showing stronger correlations among themselves, and finally, an inverse relationship between some toxic metals and essential elements.

### 3.2. Linear Regression for Association of Combined Toxic Metals and Essential Element on Liver Dysfunction

Table 2a shows that Mn has a statistically significant positive relationship with ALT, while Pb, Cd, Hg, and Se have no significant relationship with ALT. In Table 2b, Pb has a statistically significant positive relationship with AST with *p*-values of 0.019. However, Cd, Hg, Mn, and Se have no statistical relationship with the liver biomarker AST. Table 2c shows that Pb has a positive association with ALP, with *p*-values of 0.010. However, Hg and Mn have a negative relationship with ALP. Cd and Mn did not have a strong association with ALP. In Table 2d, Pb and Mn have a positive association with GGT, but Hg has a negative association with GGT. Cd and Se do not have a significant association with the GGT biomarker. For total bilirubin, only Se has a positive relationship with *p*-values of 0.006, while there was no significant relationship with Pb, Cd, Hg, and Mn (Table 2e). Table 2f shows that US-FLI has a significant positive relationship with Mn but not with Pb, Cd, Hg, and Se. The observed significant association between Mn and ALT/US-FLI levels, as determined through multivariable linear regression, was interesting given Mn’s role as an essential trace element involved in enzyme activation and antioxidative protection. The relationship is likely dose-dependent.

The coefficients in the tables represent the relationship between various toxic metals and essential elements (such as Pb, Cd, Hg, Mn, and Se) and different liver injury indicators (ALT, AST, ALP, GGT, Total Bilirubin, and US-FLI). These coefficients were calculated using regression models adjusted for covariates, including age, sex, BMI, alcohol consumption, ethnicity, income, and smoking status.

The coefficients represent the change in each liver function indicator for a one-unit increase in the exposure to the respective toxic metal or essential element. A positive coefficient indicates that as the exposure to the element increases, the level of the liver function indicator also increases. Conversely, a negative coefficient suggests that increased exposure to the element is associated with a decrease in the liver function indicator. The *p*-values indicate the statistical significance of the coefficients, with values less than 0.05 typically considered significant. The 95% confidence intervals provide a range within which the true coefficient value is likely to lie, offering insight into the precision of the estimates.

### 3.3. BKMR Results

The need to comprehensively model the interactive and non-linear effects among the variables in our dataset led to the adoption of Bayesian Kernel Machine Regression. Unlike traditional linear regression approaches, this method is particularly well suited for analyzing the potential synergistic and antagonistic interactions within complex environmental exposure data, which may not adequately capture such dynamics. Traditional linear regression operates under the assumption of linearity and independence between variables, which often does not hold in complex environmental studies where variables may interact in non-linear and dependent ways. Additionally, linear regression may not effectively handle the high dimensionality and collinearity among variables, which are common in environmental exposure data. BKMR, on the other hand, is designed to address these limitations by allowing for the exploration of complex exposure–response relationships and providing a more nuanced understanding of how mixtures of exposures impact health outcomes. This approach is especially valuable in environmental health research, where multiple correlated exposures can influence the results in subtle yet significant ways.

Bayesian Kernel Machine Regression (BKMR) stands out in its ability to handle scenarios characterized by complex and non-linear relationships among variables. Through the use of adaptable kernel functions and Bayesian inference, BKMR is adept at identifying latent patterns and accommodating interactive effects among variables, effectively capturing intricate dependencies that traditional linear regression models frequently overlook. This methodological versatility enables BKMR to deliver precise and insightful conclusions, making it an exceptionally robust analytical tool for studying multifaceted data interactions. BKMR was employed to elucidate the complex effects of toxic metals and essential elements on NAFLD.

#### 3.3.1. Analysis of Posterior Inclusion Probabilities (PIPs) for Metals and Essential Elements in Relation to NAFLD Risk

Table 3 represents the PIPs for each metal and essential element and their relationship with NAFLD. The result shows that Pb has the highest probable relationship with NAFLD, followed by Hg, Cd, Se, and Mn, which have the least relationship with NAFLD. The Posterior Inclusion Probability quantifies the relative importance of each toxic metal and essential element in explaining variations in NAFLD biomarker levels. Our results indicate that Pb and Hg, with PIP values of 1.0000 and 0.9512, respectively, are the most significant contributors to the variability in NAFLD levels, with Pb exhibiting the greatest impact.

#### 3.3.2. Univariate Analysis: Examining the Isolated Effects of Pb, Cd, Hg, Se, and Mn on NAFLD

**Figure 2:**
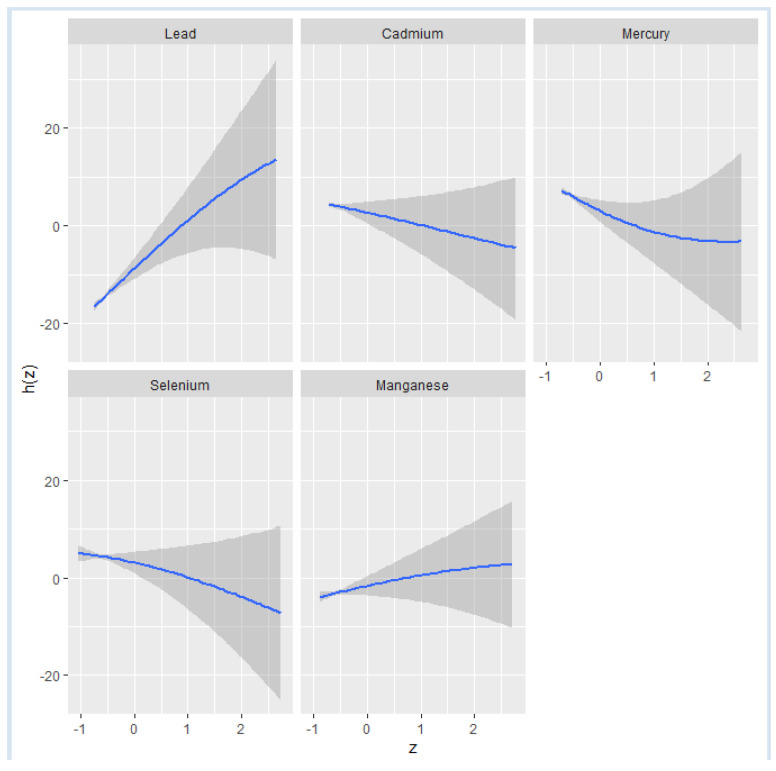
Univariate exposure–response functions and 95% confidence interval for the association between single metal and individual essential element exposure with NAFLD when other exposures are fixed at the median.

Figure 2 shows the effect of individual metals and essential elements on NAFLD when the other variables are fixed at the median and the covariates are held constant. A steady upward curve for Pb indicates a positive relationship between Pb and NAFLD. The relatively flat trend observed for the remaining exposures indicates a minimal relationship between them and NAFLD. Moreover, the single variable analysis, which examined the effects of toxic metals and essential elements, further revealed that Pb and Mn are associated with an increased risk of NAFLD. Among these, Pb exhibited the most pronounced effect, suggesting it substantially impacts the risk of developing this liver condition.

#### 3.3.3. Bivariate Exposure–Response Function of Toxic Metals and Essential Elements with NAFLD

**Figure 3:**
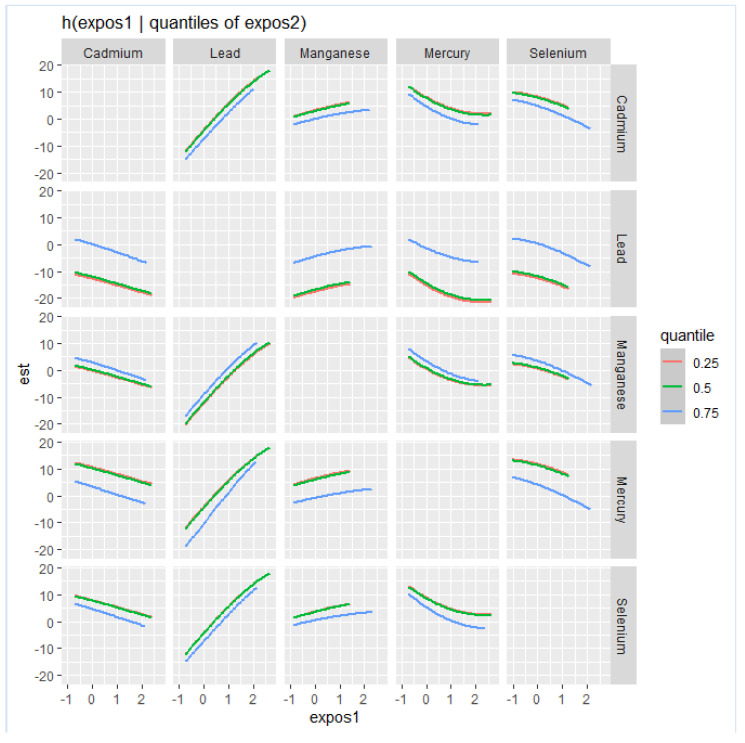
Bivariate exposure–response function of toxic metals and essential elements with NAFLD, where the argument specifies a sequence of quantiles at which to fix the second predictor.

This analysis (Figure 3) investigated the association between individual metals and essential elements on NAFLD by fixing the second metal or essential element at various quantiles (25th in red, 50th in green, and 75th in blue) while keeping other metals and essential elements at their median values. These models were adjusted for relevant covariates. The x-axis, denoted as “expos1”, displays the levels of one exposure, while the y-axis, labeled “est”, represents the estimated effect on NAFLD levels. Each row of plots corresponds to a different exposure considered as “expos1”.

Cd (as expos1): When interacting with Cd, the effects on NAFLD appear to be relatively flat at all quantiles of Pb, Mn Hg and Se, suggesting that the impact of Cd on NAFLD levels is minimal.

Pb (as expos1): The plots for Pb show a robust positive relationship with NAFLD at all quantiles of Cd, Mn, and Hg and at the 50th and 75th quantiles for Se.

Mn (as expos1): the interaction for Mn plots shows a relatively flat relationship with NAFLD at different quantiles of Cd, Pb, Hg, and Se.

Hg (as expos1): Hg’s interaction plots show a relatively flat relationship with NAFLD at different 25th, 50th, and 75th quantiles of Cd, Pb, Mn, and Se.

Se (as expos1): the plots for the interactions for Se reveals a relatively flat relationship with NAFLD at different quantiles of Cd, Pb, Mn, and Hg.

Effect of quantiles: Variations in the shapes of the lines across different quantiles of “expos2” within each plot illustrate how the impact of “expos1” on NAFLD fluctuates with different levels of “expos2”. For example, in the interaction between Pb and Mn, the curves for the 25th, 50th, and 75th quantiles of Mn are relatively the same, suggesting consistent effects at lower to mid-level to higher levels of Mn.

### 3.4. Overall Risk Summary of NAFLD Levels in Relation to Exposure Percentiles

**Figure 4:**
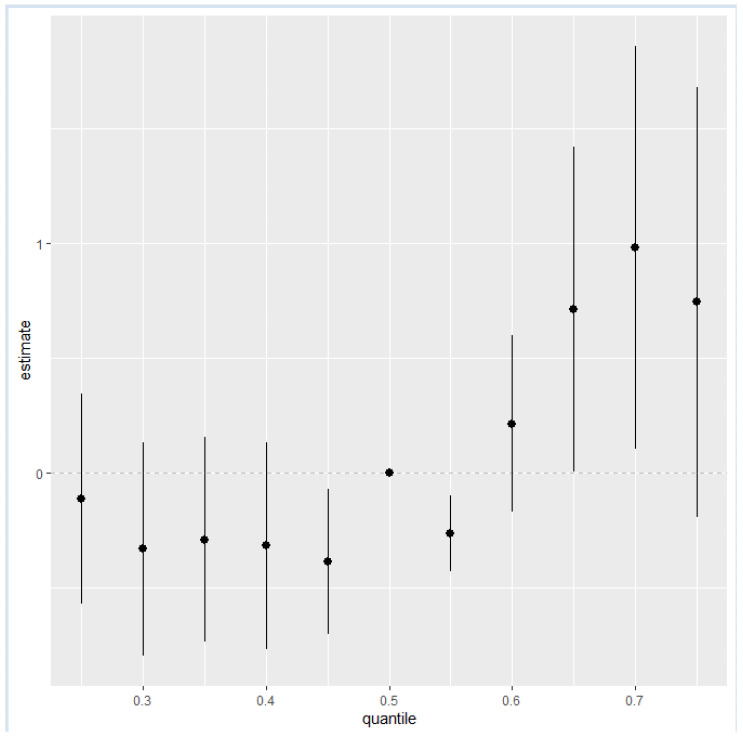
The overall health effects of the exposures calculated by comparing the value of h when all of predictors are at a particular percentile as compared to when all of them are at their 50th percentile.

Figure 4 quantifies the cumulative effect of all exposures or mixtures. Exposures are held constant at various percentiles ranging from the 25th to the 75th percentile, increasing by increments of 5, with the 50th percentile (median) serving as a reference for comparison. As shown in the plot, the estimation risk of NAFLD for all exposures from the 25th to the 55th percentile was slightly below zero except for the 50th percentile, which was exactly zero; however, there was a steady increase from the 60th percentile to the 75th percentile. This trend underscores the importance of monitoring and managing combined exposures to toxic metals and essential elements to mitigate the risk of NAFLD, particularly as exposure levels increase beyond the certain thresholds.

### 3.5. Single-Variable Effects of Toxic Metals and Essential Elements on NAFLD

**Figure 5:**
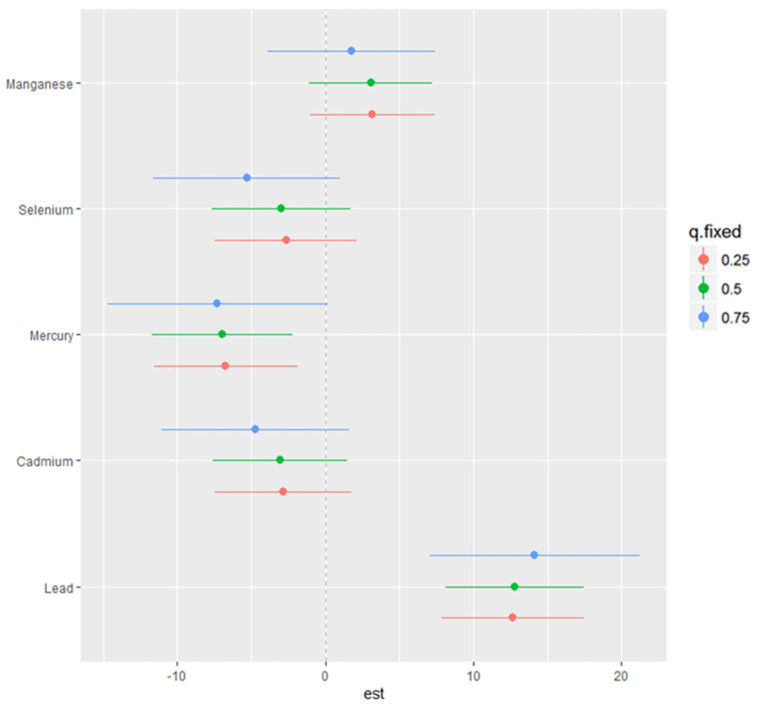
Single-variable interaction effect of toxic metals and essential elements at increasing quantiles of NAFLD.

The examination of single-variable effects aids in comprehending how a single predictor influences NAFLD levels across various quantiles, allowing for the evaluation of their respective contributions to the overall risk of elevated NAFLD. Figure 5 illustrates the single-variable effects of toxic metals and essential elements on NAFLD at the 25th (red), 50th (green), and 75th (blue) percentiles, indicating that Pb is most associated with higher values of the *h* function, a flexible function that incorporates multiple toxic metals and essential elements and integrates them in a manner that captures the intricate and potentially non-linear correlation between the toxic metals, essential elements and NAFLD. Specifically, the results illustrate that Pb has a significant positive association with NAFLD, particularly at the 75th percentile, indicating that higher Pb exposure is associated with an increased risk of NAFLD at higher levels of the disease. Mn also shows a positive association across all percentiles, reinforcing its complex role in liver function and potential contribution to NAFLD. Se has a more variable association, with significant effects at certain percentiles, suggesting it may have a nuanced role in NAFLD development. These findings highlight the importance of considering individual toxic metals and essential elements in the risk assessment and management of NAFLD.

## 4. Discussion

NAFLD is a growing health concern in the United States and worldwide [3]. Exposure to toxic metals can negatively impact liver health and increase the risk of developing NAFLD [13]. Conversely, imbalances or deficiencies in essential elements can also contribute to the development of NAFLD [12]. Our study sought to understand the intricate relationship between exposure to both toxic metals and essential elements on liver dysfunction. Toxic metals interfere with the body’s absorption, excretion, and transport of essential metals, as well as with their binding to target proteins and their metabolism and sequestration [28]. For instance, Pb toxicity partly arises from its ability to mimic essential metals like zinc, binding to and interacting with many of the same enzymes, thereby disrupting normal enzymatic reactions [29,30]. Similarly, Cd and Pb share chemical and physical properties with zinc and compete for the binding sites on metal absorptive proteins and enzymes [31]. In situations of zinc deficiency and heightened exposure to Cd and Pb, the body may utilize these toxic metals as substitutes for zinc [10].

Our analysis began by exploring this relationship via linear regression. The observed significant association between Mn and ALT levels, as determined through multivariable linear regression, was interesting given Mn’s role as an essential trace element involved in enzyme activation and antioxidative protection. The relationship is likely dose-dependent [32], as Mn is beneficial in trace amounts but may become hepatotoxic at elevated concentrations. High levels of Mn could disrupt hepatic function by promoting oxidative stress, provoking inflammatory responses, or interfering with the metabolic processes of other essential minerals and nutrients [33]. This correlation might not signify direct causality but rather suggests that elevated Mn levels could be an indicator of exposure or a marker of metabolic dysregulation, potentially linked to hepatic stress or injury [34]. The significant associations identified between Pb and aspartate aminotransferase (AST) levels through multivariable linear regression warrant careful interpretation and further investigation. AST is an enzyme localized in the liver and found in the heart, muscles, and brain, where it plays pivotal roles in amino acid metabolism and the generation of cellular energy [35]. The presence of AST in multiple organs suggests that elevated blood levels could indicate damage to the liver and other tissues.

Pb exposure’s link to increased AST levels could imply multi-organ risk, as Pb is known to disrupt several biological systems [36]. The relationship observed might reflect Pb’s capacity to induce oxidative stress, disrupt mitochondrial function, or interfere directly with cellular enzymes, potentially leading to tissue damage and increased enzyme leakage into the bloodstream [37]. Given AST’s broad tissue distribution, its elevation in response to Pb exposure might serve as a useful biomarker for systemic toxicity [38]. When analyzed alongside ALT, AST offers valuable insights into the underlying causes of liver disease [34].

The multivariable linear regression analysis revealing both positive and negative associations of specific metals/elements with alkaline phosphatase (ALP) levels presents a complex scenario that necessitates detailed exploration. ALP comprises a group of isoenzymes localized on the external surface of cell membranes, primarily involved in catalyzing the hydrolysis of organic phosphate esters in the extracellular space [39]. The positive associations found between Pb and Mn with ALP levels suggest an upregulation or increased expression of these isoenzymes, potentially as a response to metal-induced stress or damage to cellular membranes.

Conversely, the negative association observed with Hg implies a possible inhibitory effect on ALP activity, which could reflect disruptions in cellular membrane integrity or enzyme function [39]. These differential impacts highlight each metal’s distinct biological interactions with cellular processes, particularly those involving membrane-bound enzymes. Given ALP’s role in a variety of physiological processes, including bone mineralization and liver function, the implications of altered ALP activity due to metal exposure are significant [40].

Mn is crucial for various biological functions, including enzyme activation and antioxidative processes [41], yet, as observed in this study, it also exhibits a positive correlation with gamma-glutamyl transferase (GGT) levels, which might suggest a potential dual role depending on its concentration and physiological context. The beneficial roles of Mn at optimal levels contrast with its potential for toxicity at higher concentrations [42], which might induce hepatic stress. This stress could manifest as elevated GGT levels, reflecting Mn’s impact on the liver when present in excess. Similarly, the study found a positive association between Pb and GGT levels, indicating that Pb exposure could also be contributing to liver dysfunction, as evidenced by the elevated enzyme levels [43]. In contrast, Hg showed a negative association with GGT, suggesting a possible inhibitory effect on the enzyme, which could reflect a different toxicological impact on liver function. Thus, while Mn and Pb may contribute to increased GGT levels and subsequent liver dysfunction when in excess, Hg’s interaction appears to suppress GGT activity, each reflecting distinct modes of hepatic influence by these metals [44].

In the context of this study’s multivariable linear regression analysis, Se displayed a slight but significant positive association with total bilirubin levels. Bilirubin is a byproduct of the breakdown of red blood cells and is primarily processed by the liver for excretion from the body. Normally, bilirubin levels in the blood are kept low through efficient processing and excretion by the liver [45]. However, elevated levels of total bilirubin are often indicative of liver dysfunction or impairment, as they suggest a disruption in the liver’s ability to process and clear bilirubin effectively. The observed association between Se and bilirubin levels introduces the possibility that Se might influence liver function in complex ways despite its essential roles as an antioxidant and in thyroid hormone metabolism. This slight positive correlation could reflect Se’s involvement in subtle biochemical pathways in the liver that affect bilirubin metabolism, or it could indicate that elevated Se levels, potentially above optimal thresholds, impact liver health [46,47].

In the results of the multivariable linear regression analysis conducted in this study, there was a significant positive association between Se and the fatty liver index, a predictive score used to estimate the risk of steatosis in the liver. Se, known for its critical roles in antioxidant defense and immune function, typically benefits health when consumed in required amounts. However, this association suggests that elevated Se levels may be linked to an increased risk of fatty liver disease [48,49].

BKMR was employed to elucidate the complex effects of toxic metals and essential elements on NAFLD. The Posterior Inclusion Probability quantifies the relative importance of each toxic metal and essential element in explaining variations in NAFLD biomarker levels. Our results indicate that Pb and Hg, with PIP values of 1.0000 and 0.9512, respectively, are the most significant contributors to the variability in NAFLD levels, with Pb exhibiting the greatest impact. Prior work has demonstrated the dominant impact of Pb in a mixture of PFAS and metals [50]. The univariate analysis within the BKMR framework particularly highlighted the significant toxic impact of Pb, reaffirming its prominent influence among the mix of toxic and essential elements. This finding emphasizes Pb’s detrimental role when considered individually in the environmental mixture, supporting the hypothesis that Pb exposure is a critical factor in liver disease risk [51,52].

Moreover, the single-variable analysis, which examined the effects of toxic metals and essential elements, further indicated that Pb and Mn are associated with an increased risk of NAFLD. Among these, Pb exhibited the most pronounced effect, suggesting it substantially impacts the risk of developing this liver condition. These results underline the importance of considering individual metal exposures within a mixture in environmental health research, as they can distinctly influence the pathogenesis of diseases like NAFLD. This nuanced understanding helps pinpoint specific public health interventions and regulatory measures aimed at reducing exposure to hazardous substances like Pb, thereby mitigating their health impacts.

The bivariate exposure–response functions explored shed additional light on the potential for synergistic interactions between the different toxic metals and essential elements on NAFLD. Our result showed positive interactions between Pb and Cd, Pb and Mn, Pb and Hg, and Pb and Se. By examining the exposure–response patterns in a bivariate framework, we were able to uncover evidence suggesting that the combined presence and interactions would be expected from the individual metal exposures alone [53]. The more pronounced effect of combined exposures with Pb once again suggested its dominant role in influencing the manifestations of mixture exposures involving Pb.

Our analysis of the overall risk summary of NAFLD in relation to exposure percentiles revealed an increase in the estimated risk of NAFLD for all exposure from the 60th percentile to the 75th percentile, suggesting that higher exposure levels to the toxic metals and essential elements within the 60th and 75th percentile range are associated with an increased risk of developing NAFLD. The trend for the entire graph was upward, providing insight into how continuous exposure to multiple potential contaminants can adversely affect liver health [54,55].

Investigating the relationship between toxic metals and essential elements on NAFLD holds significant implications for comprehending the intricate interaction between environmental exposures and liver health. Our findings on the liver biomarkers concerning toxic metals and essential elements also give insight into the potential mechanisms by which environmental exposures may contribute to the development and progression of NAFLD [56]. Furthermore, identifying metal–element interactions and their combined influence on NAFLD risk can inform the formulation of tailored strategies for managing liver disease.

### Limitations

This study faces several limitations that merit consideration. First, the temporality issue inherent in the cross-sectional design prevents the establishment of causality between exposures and outcomes, as both are assessed simultaneously. This makes it challenging to ascertain whether exposure to toxic metals and essential elements precedes the onset of liver conditions such as NAFLD. Second, exposure misclassification is a significant concern, given that reliance on single-time point measurements may not effectively capture long-term or chronic exposure levels. Variations in individual exposure over time are not accounted for, potentially leading to inaccurate estimations of the associations between metal exposure and liver health outcomes.

Additionally, this study’s findings may suffer from limited generalizability due to the specific population sample used. The demographic characteristics, geographical location, and environmental contexts specific to the study cohort may not represent broader non-United States populations, thereby restricting the applicability of the results to other groups or settings.

Nevertheless, despite these limitations, this study offers critical insights into the combined effects of toxic metals and essential elements in liver dysfunction. It highlights important associations and potential public health implications, providing a valuable foundation for future research and interventions aimed at mitigating liver health risks associated with environmental exposures.

## 5. Conclusions

In conclusion, the complex relationship between exposure to toxic metals and imbalances in essential elements is a critical area of study in liver disease research. While the harmful effects of toxic metals like Pb, Cd, and Hg on liver health have been well studied, their interactions with essential elements such as Se and Mn, can significantly influence disease development and progression. Increasing evidence indicates that exposure to toxic metals can disturb the balance of essential elements in the body, leading to oxidative stress, inflammation, and endothelial dysfunction, which are key factors in the development of liver diseases. Conversely, maintaining optimal levels of essential elements may offer protective effects by mitigating the adverse impacts of toxic metals through their antioxidant and anti-inflammatory properties. By comprehending these mechanisms, healthcare practitioners and policymakers can proactively work towards enhancing cardiovascular well-being and alleviating the global burden of NAFLD.

This study broadens our understanding of the intricate interactions and mechanisms by which environmental metals can influence the risk of NAFLD and potentially contribute to the etiology of chronic and malignant diseases. It underscores the utilization of BKMR in environmental health research. It also reinforces the usefulness of ongoing research into the effects of combined PFAS and metal exposures on critical health outcomes. Finally, our study further closes the gap in the literature on multiple environmental pollutants exposure and health, advocating for a holistic approach to exposure assessment and risk management to improve and protect public health.

## Figures and Tables

**Table 1:** (a). Mean age by sex. (b). Mean age by ethnicity.

(a)				
Sex	Mean	Linearized Std. Error	(95% Conf. Interval)	
Male	37.427	0.493	36.375	38.478
Female	39.378	0.592	38.116	40.640
**(b)**				
**Ethnicity**	**Mean**	**Linearized Std. Error**	**(95% Conf. Interval)**	
Mexican American	29.319	0.852	27.504	31.134
Other Hispanic	33.685	0.900	31.766	35.604
Non-Hispanic White	41.670	0.785	39.996	43.343
Non-Hispanic Black	35.830	0.540	34.679	36.982
Non-Hispanic Asian	37.926	0.938	35.925	39.927
Other Race—Including Multi-Racial	33.475	1.822	29.591	37.359

**Table 2:** (a). Association between ALT and combined toxic metals and essential elements exposure. (b). Association between AST and combined toxic metals and essential elements exposure. (c). Association between ALP and combined toxic metals and essential elements exposure. (d). Association between GGT and combined toxic metals and essential elements exposure. (e). Association between total bilirubin and combined toxic metals and essential elements exposure. (f). Association between fatty liver index and combined toxic metals and essential elements exposure.

(a)					
ALT	* Coefficient	Linearized Std. Error	*p*-Value	(95% Conf. Interval)	
Pb	0.355	0.433	0.425	−0.568	1.277
Cd	0.101	0.924	0.915	−1.869	2.071
Hg	−0.401	0.344	0.262	−1.133	0.332
Mn	0.364	0.120	0.005	0.130	0.598
Se	0.040	0.022	0.086	−0.006	0.086
**(b)**					
**AST**	* Coefficient	**Linearized Std. Error**	***p*-Value**	**(95% Conf. Interval)**	
Pb	0.908	0.347	0.019	0.169	1.647
Cd	0.577	1.268	0.656	−2.125	3.279
Hg	−0.197	0.232	0.409	−0.691	0.297
Mn	0.324	0.153	0.052	−0.003	0.652
Se	−0.004	0.016	0.819	−0.039	0.031
**(c)**					
**ALP**	* Coefficient	**Linearized Std. Error**	***p*-Value**	**(95% Conf. Interval)**	
Pb	2.509	0.853	0.010	0.690	4.327
Cd	0.480	1.592	0.767	−2.914	3.875
Hg	−1.258	0.453	0.014	−2.223	−0.292
Mn	0.650	0.304	0.050	0.001	1.299
Se	−0.059	0.028	0.048	−0.118	−0.000
**(d)**					
**GGT**	* Coefficient	**Linearized Std. Error**	***p*-Value**	**(95% Conf. Interval)**	
Pb	3.287	1.067	0.008	1.013	5.561
Cd	4.411	2.861	0.144	−1.686	10.509
Hg	−0.890	0.399	0.041	−1.741	−0.041
Mn	1.205	0.361	0.005	0.435	1.976
Se	0.010	0.030	0.738	−0.053	0.074
**(e)**					
**Total Bilirubin**	* Coefficient	**Linearized Std. Error**	***p*-Value**	**(95% Conf. Interval)**	
Pb	−0.000	0.005	0.987	−0.011	0.011
Cd	−0.012	0.008	0.142	−0.028	0.004
Hg	0.005	0.007	0.458	−0.009	0.019
Mn	−0.000	0.003	0.896	−0.006	0.005
Se	0.001	0.000	0.006	0.000	0.002
**(f)**					
**US-FLI**	* Coefficient	**Linearized Std. Error**	***p*-Value**	**(95% Conf. Interval)**	
Pb	0.800	0.768	0.315	−2.435	0.838
Cd	2.380	1.359	0.100	−0.515	5.276
Hg	0.049	0.349	0.890	−0.696	0.794
Mn	0.478	0.211	0.039	0.028	0.929
Se	0.035	0.025	0.179	−0.018	0.087

* Adjusted for covariates age, sex, BMI, alcohol consumption, ethnicity, income, and smoking status.

**Table 3:** Posterior Inclusion Probabilities for the influence of toxic metals (Pb and Cd), and essential elements (Hg, Se, and Mn) on NAFLD.

Variable	PIP
Pb	1.0000
Hg	0.9512
Cd	0.5796
Se	0.5572
Mn	0.4248

## Data Availability

The NHANES dataset is publicly available online, accessible at https://wwwn.cdc.gov/nchs/nhanes/continuousnhanes/overview.aspx?BeginYear=2017 (accessed on 22 December 2023).
